# Type 1 diabetes care: Improvement by standardization in a diabetes rehabilitation clinic. An observational report

**DOI:** 10.1371/journal.pone.0194135

**Published:** 2018-03-12

**Authors:** Helmuth Haslacher, Hannelore Fallmann, Claudia Waldhäusl, Edith Hartmann, Oswald F. Wagner, Werner Waldhäusl

**Affiliations:** 1 Department of Laboratory Medicine, Medical University of Vienna, Waehringer Guertel 18–20, Vienna, Austria; 2 Rehabilitation Clinic for Diabetes and Metabolic Diseases, Moorbad Neydharting, Neydharting 4, Bad Wimsbach-Neydharting, Austria; 3 Department of Radiotherapy, Medical University of Vienna, Waehringer Guertel 18–20, Vienna, Austria; 4 Department of Medicine III, Medical University of Vienna, Waehringer Guertel 18–20, Vienna, Austria; Jimma University, ETHIOPIA

## Abstract

**Background:**

T1D treatment requires informed self-responsible patients, who, however, frequently miss their therapeutic goals, providing considerable potential for improvement.

**Methods:**

This observational report evaluates T1D patients [N = 109], aged ≥18 years (range 22–82), poorly controlled at home, at and 3 weeks after their admission to our diabetes rehabilitation clinic [DRC], where they were offered standardized, but unmonitored life-style modification.

**Results:**

At admission, patients displayed elevated HbA_1c_ values (66 mmol/mol [57; 81]), a high prevalence of co-morbidities (88%), lipodystrophies due to monolocal insulin injections (42%), a low rate of influenza (16%) and pneumococcal (7%) immunization, and underuse of lipid-lowering drugs (-38%). Standardization of life-style improved glucose (p<0.0001) and lipid metabolism (LDL/HDL ratio p<0.01) permitting reduction of insulin dose and reduction of add-on glucose-lowering drugs (GLDs) other than metformin. Outcome was independent of the mode of insulin treatment strategy and more marked at initially high HbA_1c_, with DRC-costs/d less than 25% of those encountered at standard hospitals.

**Conclusion:**

Type 1 diabetes care requires *i)* insulin treatment, food intake and life style to be handled in concert, *ii)* this need cannot be replaced by arbitrary addition of add-on GLDs, and *iii)* training to this end is 75% cheaper at a DRC than in standard hospitals.

## Introduction

In the absence of a cure, insulin replacement is mandatory for survival in patients with type 1 diabetes mellitus and requires them to be thoroughly informed and trained. They not only have to learn how to replace insulin properly [1], but also to self-administer treatment reliably, as they need to care for themselves alone between visits to their doctor commonly at intervals of 6 to 12 weeks. To shoulder the task of being ‘his own nurse, doctor’s assistant, chemist’ [2] and even physician, and to meet the metabolic goals set [3, 4], patients have to continuously maintain motivation for multiple daily interventions. These include insulin injections and blood glucose [BG] self-measurements to keep their BG within the desired range, i.e. HbA_1c_ below 53 to 58 mmol/mol, if insulin reactions [4] and diabetes-related complications are to be avoided [5]. To provide comprehensive self-care type 1 diabetes patients are also required to document BG values, food intake, administered insulin doses, and bouts of exercise [6, 7], as well as blood pressure (BP), which taken together, may well overstretch individual motivation to comply.

The clinical outcome of this therapeutic approach is variable at best, as type 1 diabetes patients’ HbA_1c_ values, a surrogate marker of long-term BG concentration, fluctuate widely. Thus, set goals are met by a minority only (18%), while 37% even display values >64 mmol/mol (67±18 mmol/mol) [8], which is similar to the 72±23 mmol/mol seen in type 1 diabetes patients 30 years ago in rural area [9] and potentially worsening over time [10]. The associated annual economic costs range in central Europe from € 5,331 (Switzerland 2014) to € 5,899 (Germany 2010) per diabetic patient [11, 12]. The development of novel, cost-effective treatment strategies, as e.g. the integration of lifestyle interventions, is therefore highly warranted. However, only a few studies are available reporting the impact of life style on required medication other than insulin and metabolic parameters in type 1 diabetes patients. This is associated with the odd situation that most standard recommendations on physical activity for T1D patients are based on data from healthy subjects or from patients with T2D [13].

If patient-centered self-care is to be more than rhetoric, its outcome consequently has to be routinely measured by providers of care [14, 15] to identify its potential for metabolic improvement and reduction of costs. This observational study explores besides associated costs and risk factors (smoking, depression, compliance with influenza and pneumococcal immunization), vital variables, metabolic outcome, and medications in type 1 diabetes patients at time of their admission vs. that at discharge after a 3-week stay at our Diabetes Rehabilitation Clinic (DRC).

## Methods

### Study patients

This single-center, explorative study determines improvement in outcome of diabetes care in consecutively admitted type 1 diabetes patients (N = 109), aged ≥18 years (range 22–82), who did not meet their therapeutic goals at home, by identifying changes in selected vital and metabolic variables in response to a 3-week stay at our DRC vs. those observed at admission. The study was approved by the Ethics Committee of the Medical University of Vienna (#1527/2016), and all patients included provided written, informed consent to participate.

Type 1 diabetic patients were referred to the DRC via the Austrian insurance system because of inadequate metabolic control at home, where they were attended either by general practitioners [GP] or by diabetes outpatient services [DOS]. Inclusion in the study required the referring diagnosis to be supported by at least two of the following indicators: i) age at diagnosis <40 years, ii) body mass index [kg/m^2^] <30, or a description as being a slender or non-obese adult at that time, iii) insulin treatment from onset of disease or serum C-peptide <0.8 ng/ml with blood glucose >4.4 mmol/l at any time, and/or iv) positive GAD-65 antibodies at any time after onset of hyperglycaemia [8], which reduced the number of type 1 diabetes patients included by six to 109.

### Treatment

Patients were offered a structured refresher course on diabetes self-care tailored to their individual treatment mode [1] and medical counseling, while their adherence to treatment recommendations remained unmonitored and was only subject to control by local peer pressure.

Insulin treatment strategies. Conventional insulin therapy (CIT) is defined as predetermined dosing and timing of either premixed insulin or long- and short-acting insulin injections accompanied by agreed timing of a defined food and exercise load. Functional insulin therapy (FIT; basis/bolus; physiologic treatment) is defined as disconnected administration of basal (long-acting) and prandial (short-acting) insulin, with individual dosing of the latter as deemed appropriate by the educated patient to compensate for meals or hyperglycaemia, ensuring maximum patient autonomy [1, 5]. Any mix of components of CIT and FIT is termed Intensified insulin treatment (IIT). Both FIT and IIT were administered either by multiple insulin injections or with an insulin pump (CSII, Continuous subcutaneous insulin infusion).

Intake of non-insulin glucose lowering drugs (GLDs) is documented as number of tablets/doses of metformin, DPP4 inhibitors, metformin combined with DPP4 inhibitors, SGLT-2 inhibitors, or GLP-1 receptor agonists administered per day. Additional medication is documented as number of tablets ingested per day for lipid lowering drugs (statins), antihypertensive (ACE inhibitors, angiotensin II receptor blockers [ARBs], diuretics, calcium antagonists, beta-blockers, or alpha-blockers) and antidepressant drugs, and for any other medication.

Life-style. Throughout their stay at our DRC, type 1 diabetes patients were exposed to a standardized, but unmonitored lifestyle offering mixed food, rich in vegetables and fruits providing three meals totaling 1,200 to 2,000 kcal/d depending on BMI, plus an exercise load, such as hiking, swimming and gymnastics, equivalent to an energy expenditure of 400 to 600 kcal/d.

### Examinations

Medical history documented standard information, co-morbidities, frequency of past hypoglycaemic events (symptomatic or incidental BG <2.8 mmol/l; N/week), smoking habits and compliance with recommendations for influenza and pneumococcal immunization [16].

Physical examination documented body weight and height, BMI (body mass index, weight [kg]/height [m^2^]), waist circumference [cm] and blood pressure (BP [mmHg]) as well as lipodystrophies and/or infiltrations at insulin injection sites. Diabetic neuropathy was rated using a neuropathy symptom (NSS) and deficit (NDS) score ranging from 0 (normal) to 10 (severe) [17].

### Laboratory tests

Laboratory analyses were performed at the MVZ für Laboratoriumsmedizin, Raubling GmbH, Germany using ISO 15189 accredited standardized procedures and are presented in SI units. Glomerular filtration rate (GFR, ml/min) was estimated using the CKD-EPI equation [18]. HbA_1c_ measurements ([mmol/mol]; HPLC, Bio-Rad Variant II; Bio-Rad Laboratories Inc., Hercules, USA) were made at intervals of three weeks as its changes, though smaller, can already be detected as early as two weeks after an intervention [19].

### Statistical analyses

Continuous data are given as means ± SD or median (interquartile range, IQR), categorical data as counts and percentages. Comparisons of paired continuous data were made using the non-parametric Wilcoxon test, or, where appropriate, Student's t-test. Correlations between continuous data were calculated according to Pearson (linear) and Spearman (non-linear). Independent data were compared by ANOVA and post-hoc t-tests and categorical data by Pearson’s χ^2^ tests, whereas deviations of dichotomous variables from uniform distributions were assessed by binomial tests.

Outcome was evaluated by interpreting main effects and interactions derived from general linear models with repeated measurement design (SPSS [IBM, Armonk, NY, USA]).

Analytically relevant differences between HbA_1c_ concentrations at baseline and discharge are defined as changes exceeding the reference change value [20]:

$$RCV=Z*\sqrt{2*({CV}_{A}^{2}+{CV}_{I}^{2})}$$

Given a Z (Z-score) of 1.96 for significance at 95% probability level, a CV_A_ (analytical coefficient of variance) of 1.05% and a CV_I_ (individual biological variation) of 1.9% [21], a relative difference >6% in HbA_1c_ concentration between baseline and discharge was considered diagnostically relevant.

Possible decreases in HbA_1c_ were predicted by binary logistic regression models, whereas goodness of fit was evaluated by interpreting areas under the curve (AUC) of receiver operator characteristic (ROC) plots. p-Values, recalculated according to Benjamini and Hochberg, were considered significant if <0.05.

Improvements of HbA_1c_, LDL, and mean arterial blood pressure (MAP, diastolic BP plus BP-amplitude/3) were determined after three weeks as reduction at discharge vs. values at admission, and expressed as percentage of their respective initial deviation from ADA benchmarks (HbA_1c_, 53 mmol/mol; LDL, 1.81 mmol/mol and MAP, <107 mmHg) [3].

Figures were drawn using GraphPad Prism 6 (GraphPad Software Inc., La Jolla, Ca, USA), MedCalc version 15.8 (MedCalc Software bvba, Ostend, Belgium), or SPSS 23 (IBM), which was also used for calculations.

## Results

### Study population

Baseline characteristics of type 1 diabetes patients are presented in Tables 1 and 2. In brief, we registered a preponderance of males (54%), with a median duration of disease of 18 years and a high frequency of lipodystrophies and infiltrations at monolocal insulin injection sites (42%). Insulin demand was 15 percent greater at admission (0.53 [0.44; 0.65] U/kg b.w. [body weight]) than at the time of discharge (0.46 [0.39; 0.53] U/kg b.w.; p<0.0001), when infiltrated areas were avoided. Hypoglycaemia was encountered once/week and its rate not different between sexes, while nephropathy (GFR < 60 ml/min) was seen in 9 per cent of patients.

**Table 1:** Baseline characteristics of T1D patients (N = 109) segregated for females and males.

	Median (IQR) or counts (%)		p-Value
	male	female	
**Sex**	59 (54%)	50 (46%)	n.s.
**Age (years)**	51 (38; 55)	48 (43; 55)	n.s.
**Medical history**			
** - Duration of type 1 diabetes, years**	20 (12; 30)	18 (7; 27)	n.s.
** - Smokers**			
** - active**	23 (39%)	17 (34%)	n.s.
** - former**	8 (14%)	7 (14%)	
** - Vaccination**			
** - influenza**	9 (16%)	8 (16%)	n.s.
** - pneumococci**	5 (9%)	3 (6%)	n.s.
**Diabetes-associated complications**			
** - Lipodystrophy/Infiltrations**	27 (54%)	19 (44%)	n.s.
** - Hypoglycaemias/week**	1 (0; 2)	1 (0; 2)	n.s.
** - Neuropathy**	13 (23%)	4 (8%)	n.s.
** - NSS**	0 (0; 4)	0 (0; 3)	n.s.
** - NDS**	2 (1; 4)	2 (0; 4)	n.s.
** - Nephropathy**	6 (10%)	4 (8%)	n.s.
** - creatinine [μmol/L]**	79.6 (70.7; 97.2)	66.3 (53.0; 79.6)	<0.0001
** - GFR >90 [ml/min/1.73m^2^]**	39 (66%)	25 (50%)	n.s.
** - GFR 60–89**	15 (25%)	20 (40%)	
** - GFR 30–59**	3 (5%)	5 (10%)	
** - GFR 15–29**	1 (2%)	0 (0%)	
** - GFR <15**	1 (2%)	0 (0%)	

**Table 2:** Outcome of T1D care at admission and discharge after three weeks at the DRC (N = 109).

		Admission	Discharge	p-Value
**(a) Vital and metabolic variables**				
**- BMI (kg/m^2^)**		26.5 (23.2; 29.8)	25.8 (23.0; 29.3)	<0.01
**-Waist circumference (cm)**		96 (84; 106)	95 (83; 103)	<0.0001
**-Blood pressure (mmHg)**	**systolic**	138 (126; 151)	120 (110; 130)	<.0001
	**Diastolic**	83 (76; 93)	76 (68; 82)	<.0001
	**MAP**	102 (94;110)	90 (84; 97)	<0.001
**-HbA**_**1c**_ **[mmol/mol]**		66 (57; 81)	63 (56; 74)	<0.0001
**-Fasting serum glucose [mmol/L]**		9.7 (7.1; 11.9)	8.8 (6.9; 10.4)	<0.05
**-Total cholesterol [mmol/L]**		5.1 (4.6; 5.9)	4.3 (3.7; 5.0)	<0.0001
**-LDL cholesterol [mmol/L]**		3.1 (2.5; 3.7)	2.5 (2.0; 3.1)	<0.0001
**-Triglycerides [mmol/L]**		1.1 (0.9; 1.6)	1.0 (0.7; 1.4)	<0.001
**-LDL/HDL ratio**		1.8 (1.4; 2.6)	1.7 (1.3; 2.2)	<0.01
**(b) Medications**				
Insulin (U/(day*kg body weight))		0.53 (0.44; 0.65)	0.46 (0.39; 0.53)	<0.0001
** -long acting insulin**		0.29 (0.23; 0.38)	0.24 (0.19; 0.29)	<0.0001
** -short acting insulin**		0.25 (0.18; 0.31)	0.23 (0.18; 0.23)	<0.05
** -mixed insulin**		0.55	-	-
**Oral antidiabetics, Patients (N,%); (Tablets/d (IQR))**		21 (19%); (2 (1; 2))	21 (19%); (2 (2; 3))	n.p.
** -Glitazones**		1 (1%); (1 (-))	0 (0%); (- (-))	n.p.
** -Metformin**		12 (11%); (1½ (1; 2))	19 (17%); (2 (2; 3))	n.p.
** -Metformin plus DPP-4 inhibitor**		4 (4%); (2 (2; 2))	2 (2%); (2 (2; 2))	n.p.
** -SGLT2 inhibitors**		3 (3%); (1 (1; 1))	0 (0%); (- (-))	n.p.
** -Sulfonyl ureas**		3 (3%); (2 (1; -))	0 (0%); (- (-))	n.p.
**Hypolipidemics, Patients (N,%); (Tablets/d (IQR))**		37 (34%); (1 (1; 1))	51 (47%); (1 (1; 1))	<0.01
**Antihypertensives Patients (N,%); (Tablets/d (IQR))**		42 (39%); (1¾ (1; 3))	46 (42%); (1¾ (1; 2))	n.p.
** -ACE inhibitors**		20 (18%) (1 (1; 1))	21 (19%); (1 (¾; 1))	n.p.
** -ARBs**		12 (11%); (1 (1; 1))	12 (11%); (1 (1; 1))	n.p.
** -ARB plus diuretic**		12 (11%); (1 (1; 1))	16 (15%); (1 (1; 1))	n.p.
** -Beta-blockers**		14 (13%); (1 (1; 2))	13 (12%); (1 (½; 1¼))	n.p.
** -Calcium antagonists**		10 (9%); (1 (1; 1⅛))	6 (6%); (1 (⅞; 1⅛))	n.p.
** -Diuretics**		12 (11%); (1 (1; 2))	11 (10%); (1 (1; 1))	n.p.
**Antidepressants, Patients (N,%)**; **(Tablets/d (IQR))**		23 (21%); (1¾ (1;3))	21 (19%); (1¾ (1; 3½))	n.p.
**Others, Patients (N,%)**; **(Tablets/d (IQR))**		61 (56%); (2 (1; 3))	58 (53%); (2 (2; 3))	n.p.

n.p. … no statistical test performed.

The combined share of active (N = 40) and former smokers (N = 15) among type 1 diabetes patients was 52%, smoking a median of 20 (10; 20) cigarettes per day over 25 (17; 31) years, and did not differ between sexes. The decision of patients to stop smoking was commonly coincident with a major health hazard (stroke, myocardial infarction etc.), but never a free personal decision. Non-smokers had a higher BMI (+2.8±0.9 kg/m^2^, p<0.05), developing, similar to their waist circumference (-2.3±0.3 vs. -0.8±0.4 cm, p<0.05), more favorably at the DRC (-0.4±0.1 kg/m^2^) than in current smokers (0.0±0.1 kg/m^2^, p<0.05), who also had higher HbA_1c_ values (+8±3 mmol/mol, p<0.05).

Recommendations of influenza (16%) and pneumococcal (7%) immunization were only rarely implemented by type 1 diabetes patients.

**Fig 1:**
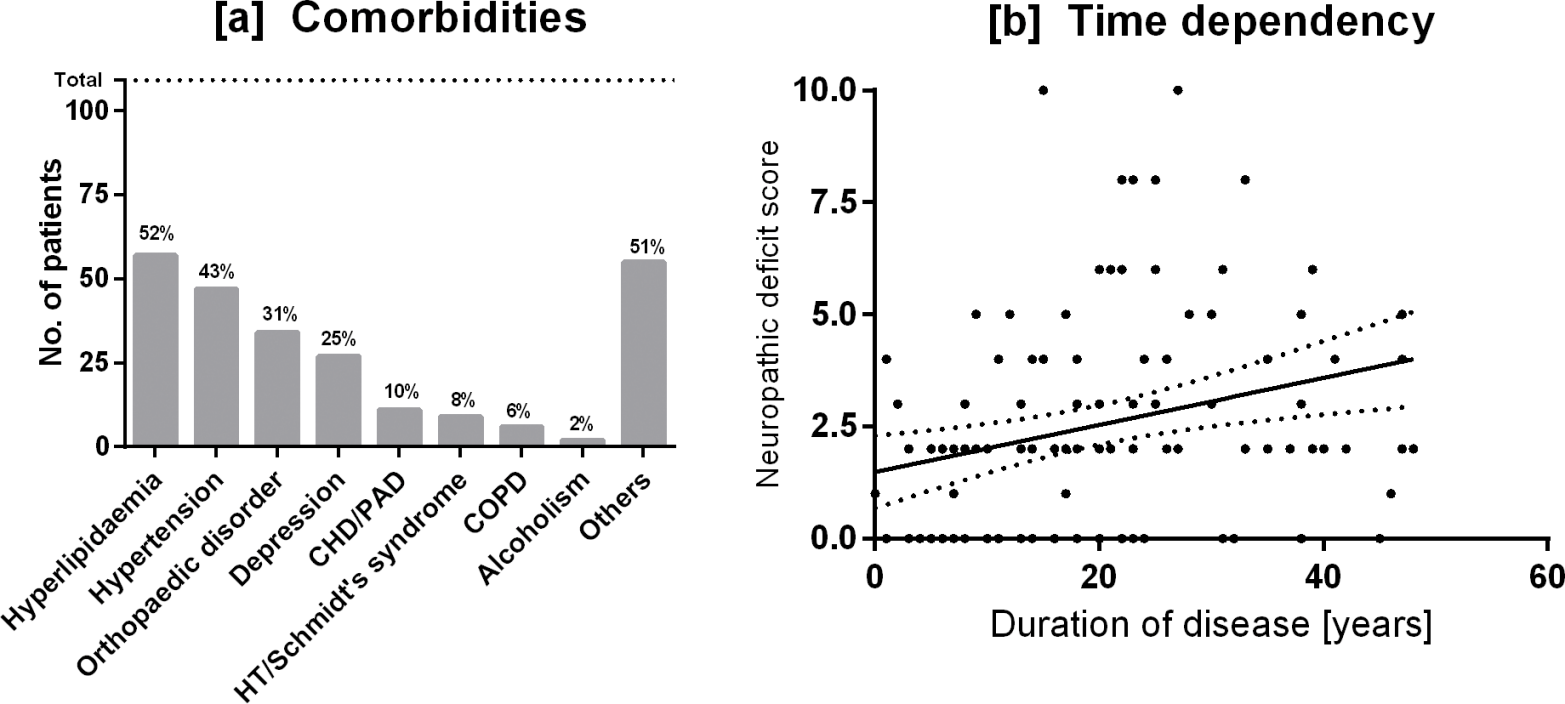
(a) Co-morbidities (N,%) in type 1 diabetes patients (N = 109). CHD, coronary heart disease; PAD, peripheral artery disease; HT, hypothyreoidism; COPD, chronic obstructive pulmonary disease, and (b) correlation of NDS (neuropathic deficit score) with duration of disease (ρ = 0.368, p<.01).

Co-morbidities (Fig 1A) were diagnosed in 88% of type 1 diabetes patients and dominated by hyperlipidaemia (52%) followed by arterial hypertension, depression, cardiovascular disease, hypothyroidism/Schmidt syndrome (8%), chronic obstructive pulmonary disease (COPD), and other disorders with a prevalence of <6% including alcoholism, carcinoma, psychosis, sleep apnea, and others.

Arterial hypertension was strongly associated with older age (52 (46; 57) vs. 46 (36; 54) years; p<0.01), increased waist circumference (+8.3±2.4 cm, p<0.01), and by trend with higher BMI (+2.0±0.9 kg/m^2^) and triglycerides (+0.29±0.13 mmol/l; both p = 0.057).

Interestingly, presence of depression neither depended on age, duration of illness, insulin dose, or lipodystrophies.

Sixteen percent of type 1 diabetes patients suffered, dependent on duration of disease (Fig 1B; ρ = 0.368, p<0.01), from diabetic neuropathy with the neuropathic deficit score (NDS, 0 [0; 3]) being somewhat smaller than the corresponding symptom score (NSS, 2 [0; 4]), demonstrating the subjective burden of complaints.

### Outcome

#### Vital and metabolic variables (Table 2A)

Analyzing clinical outcome after three weeks, significant improvement was seen in both vital (BMI, waist circumference, BP) and metabolic variables.

**Fig 2:**
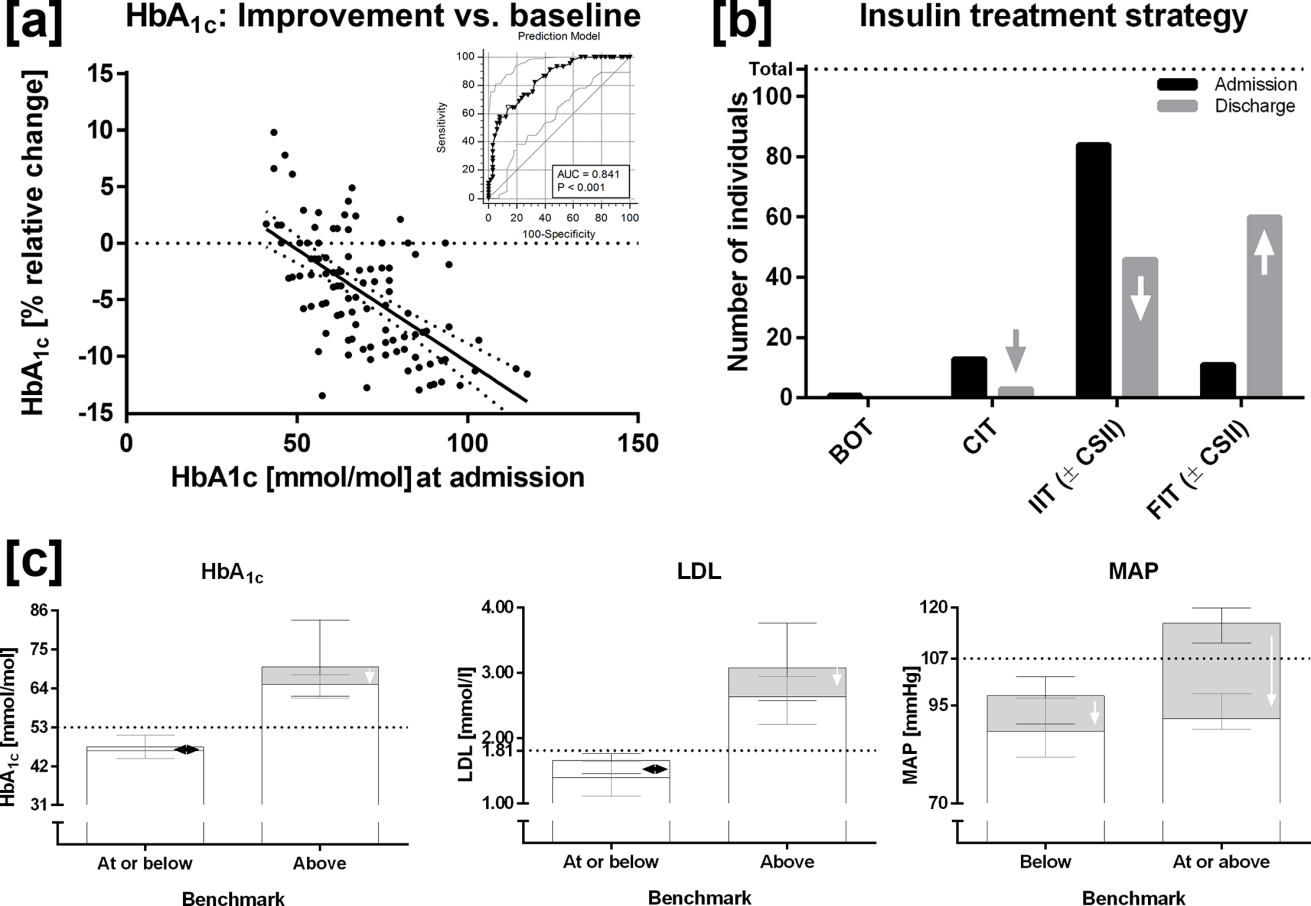
(a) Inverse correlation between HbA_1c_ values at admission and their relative changes in response to a 3-week stay at the DRC in type 1 diabetes patients (N = 109). Insert: ROC-curve from a binary logistic regression model predicting from baseline possible HbA_1c_ improvement (>6.0 relative %) in response to proper treatment. (b) Strategies of insulin treatment used by type 1 diabetes patients (N = 109) at admission and at discharge. Note the shift towards more elaborate treatment modes. BOT, Basal supported oral therapy; CIT, Conventional insulin therapy; IIT (± CSII), Intensified insulin therapy ± continuous subcutaneous insulin infusion, FIT (± CSII), Functional insulin (basis/bolus) therapy ± continuous subcutaneous insulin infusion.

At admission, 86% of type 1 diabetes patients had HbA_1c_ values above the ADA benchmark (>53 mmol/mol) and only 14% at or below the therapeutic target, with this share increasing to 17% at discharge. During the DRC stay, HbA_1c_ did not change in eight patients (8%) and even deteriorated in 17%. Of the 75% with decreasing HbA_1c_ values, 41% showed an analytically relevant change >6% from baseline, displaying an inverse linear relationship with HbA_1c_ values recorded at admission (r = -0.621, p<0.0001; Fig 2A).

Notably, the fall in serum LDL/HDL ratio did not depend on medication alone, but also on changes in lifestyle as its decline was observed in patients with pre-established (-17±31%, p<0.01) and newly established (-34±21%, p<0.0001) hypolipidaemic treatment (interaction for all three groups p<0.01). A similar pattern applied to MAP, which decreased within three weeks to the same extent in patients pretreated, newly treated and those not treated with antihypertensives (interaction p>0.05).

Of note, changes in both metabolic and vital parameters were independent from the patients’ choice of insulin treatment strategy, emphasizing the need to reliably implement pertinent treatment recommendations (see Table A and Fig A in S1 File).

Significantly, patients with initially elevated HbA_1c_ and LDL levels reduced this deviation within 3 weeks by more than thirty percent towards their respective ADA benchmark, while MAP fell even more markedly (Fig 2C).

When HbA_1c_ concentration at admission was categorized by residential postal code, some regions displayed better HbA_1c_ values (p<0.05) than others (H, 58 mmol/mol [53; 65]; <B, Δ+15±6 mmol/mol; <C, Δ+17±6 mmol/mol; <<F, Δ+21±7 mmol/mol; and <<<G, Δ+22±9 mmol/mol). In particular, G showed considerably poorer care than H, reflecting regional differences in diabetes education and care.

**Fig 3:**
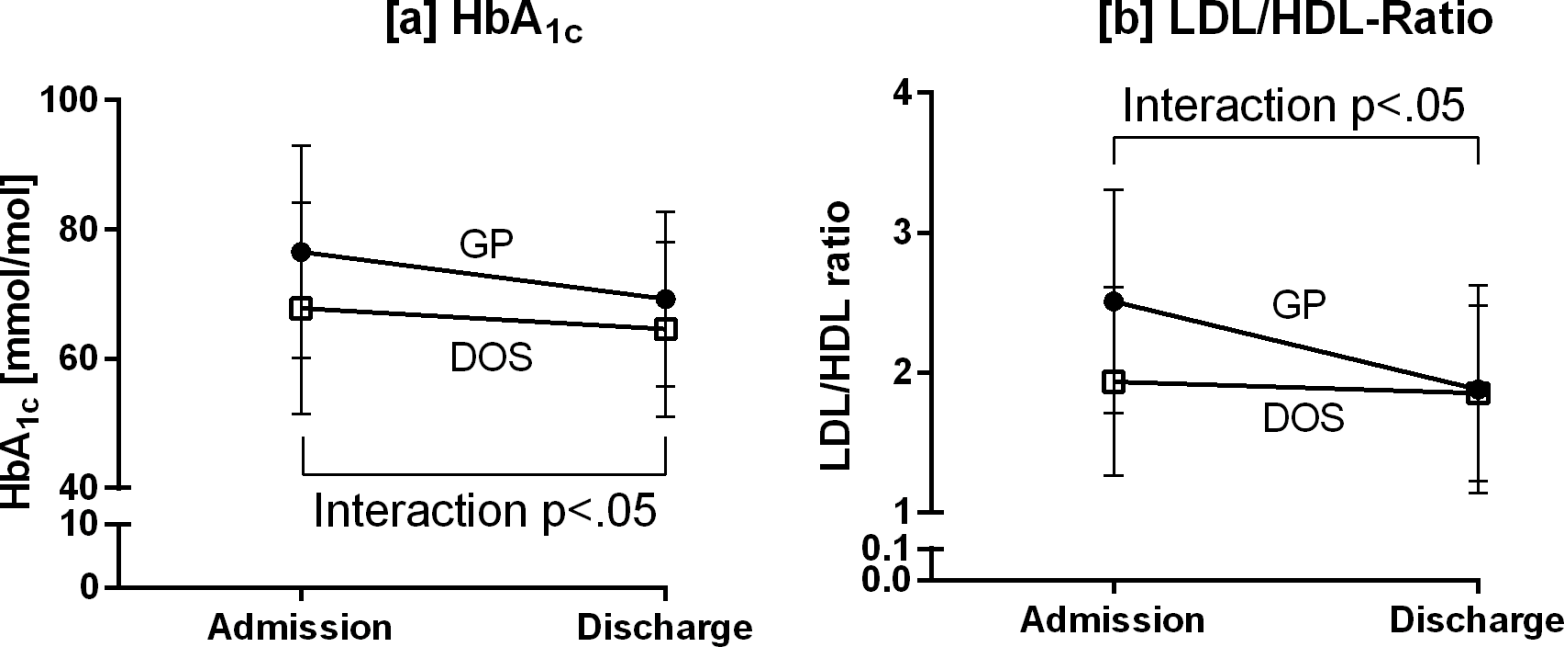
Improvement in HbA_1c_ and LDL/HDL ratio in T1D patients attended at home either by general practitioners (GP, ●) or diabetes outpatient service (DOS, □). Means ± SD.

Type 1 diabetes patients were grouped according to visits to their general practitioner (GP, 42%) or use of a specialized diabetes outpatient service (DOS, 58%) at home. As depicted in Fig 3, those seen by the former benefitted most from their stay at the DRC (HbA_1c_: GP -7±4%, p<0.0001; DOS -3±6%, p<0.01; interaction: p<0.05. LDL/HDL ratio: GP -25±23%, p<0.001; DOS -4±37%, n.s.; interaction: p<0.05).

To identify type 1 diabetes patients who might benefit most from a standardized environment we investigated potential predictors of metabolic improvement such as age, sex, BMI, waist circumference, HbA_1c_, fasting BG, total daily insulin dose, LDL/HDL ratio, and duration of disease. Of those, only baseline HbA_1c_ proved to be an independent variable predicting potential relative HbA_1c_ reductions ≥6% from baseline, exhibiting an odds ratio of 3.119 (2.011–4.838). The resulting model presented with considerable goodness of fit (Model: χ^2^ = 42.361, df = 1, p<0.0001, Nagelkerkes R^2^ = 0.434) and a good predictive capacity upon ROC analysis (AUC 0.841 (0.758–0.904)) with a maximal Youden’s index of 53% predicted probability (Fig 2A insert).

#### Medications (Table 2B)

Insulin. Median total insulin dose at admission was 40 U/d (32; 52), of which 22 (17; 30) U/d were long-acting and 18 (14; 23) U/d short-acting insulin. Although total daily insulin dose was to some extent (ρ = 0.202, p>0.05) associated with duration of disease, this correlation was nonlinear, with its first quartile (≤7 years) receiving less insulin at admission than the other quartiles (36 (32; 39) p<0.05).

To avoid hypoglycaemia due to better compliance with treatment rules at the DRC, total daily insulin dose had to be reduced by 13% from 0.53 (0.44; 0.65) to 0.46 (0.39; 0.53; p Type 1 diabetes patients with cutaneous infiltrations due to monolocal insulin injections (49%) required somewhat higher insulin doses at admission (+19%) than those without lipodystrophy (0.61 (0.47; 0.70) vs. 0.51 (0.42; 0.56) U/kg b.w., p<0.05).

At admission, most type 1 diabetes patients used some kind of intensified insulin therapy (N = 84, 77%), while only 10% employed Functional (basis/bolus) insulin therapy (FIT), 12% used Conventional insulin therapy (CIT), and only one chose basal insulin alone. Following counseling, the number of patients opting for CIT fell to three, while the majority preferred more elaborate strategies (basal/bolus 55%, IIT 42%), while the use of Continuous subcutaneous insulin infusion (CSII) rose from 17 to 26% (p = 0.002, Fig 2B).

Other medications. At admission, 85 (78%) patients received at least one additional medica-tion besides insulin. This proportion was unchanged at the time of discharge (N = 86 [79%]).

Although the number of patients receiving GLDs remained constant, add-on antidiabetic treatment shifted towards metformin, in part to avoid overinsulinzation and weight gain, replacing sulfonylureas, glitazones and SGLT-2 inhibitors (Table 2B). Such superfluous medication of GLDs other than metformin was more prevalent in type 1 diabetes patients previously attended at home by GPs than in those taken care of by specialized DOS, which also achieved better metabolic control.

The use of statins had to be increased by 38%, while that of antihypertensives remained constant for both ACE inhibitors (18%) and ARBs (22%). Their supplement with diuretics (11%), however, was replaced at discharge in part (40%) by calcium antagonists, whereas the rate of beta-blocker medication remained constant, and the use of antidepressants and other medications did not change.

### Costs

Costs per day at the DRC including board, lodging, physical rehabilitation services plus expenses for supplementary and/or modification of medication as used are modest (€131.—per day) vs. those in standard (acute) hospitals ranging from 594 to 2042 €/d (see Table B in S1 File). Of note, due to the reduction in daily insulin dose, savings, calculated as pharmacy prices, were offset by an increased need for lipid-lowering drugs, while costs for any other medication remained constant.

## Discussion

This exploratory study shows remarkable improvement of metabolic outcome if diabetes self-care is supported by standardized life-style modification, peer pressure and mutual interaction between patients at a DRC (Table 2, Fig 2A). This suggests that the outcome gap in diabetes care, i.e. the gap between diabetes control at admission and benchmark values, could possibly be narrowed or even closed at home by simple means, if type 1 diabetes patients internalized that concerted action as to insulin dosing, food intake, and exercise is requisite to therapeutic success and not merely optional.

The need to comply with respective recommendations for treatment and lifestyle is also demonstrated by the identical rate of improvement seen after three weeks at the DRC in vital (BMI) and metabolic variables (HbA_1c_, LDL/HDL ratio) independent of the applied strategy of insulin treatment.

Poor metabolic control may, however, also be due to mood swings associated with nicotine abuse [22], or to the patients’ desire to avoid hypoglycaemia as seen in males, simply by increasing carbohydrate load or reducing insulin dose [23]. Moreover, recommendations for T1D-patients as to exercise and nutrition are often drawn from type 2 diabetes-patients [24], but not evaluated as such. In this context, it is of no surprise if metaanalyses of the effect of physical exercise in addition to insulin treatment on long-term glycaemic control provide inconclusive results [25], or report only some small metabolic improvement in Type 1 diabetic children and adolescents [26–28].

Our report shows that inadequate compliance with standards of type 1 diabetes care is also commonplace with regard to i) smoking, ii) attention to co-morbidities, which ought to be treated properly as previously shown in type 2 diabetes patients [29], and iii) influenza and pneumococcal immunization [30], whose importance is frequently dismissed by both type 1 diabetes patients and by their attending physicians [31].

Any such disregard of treatment recommendations is particularly detrimental in type 1 diabetes patients, as they suffer from multiple co-morbidities. In our cohort, those were dominated by hyperlipidaemia, hypertension and obesity, the latter possibly even precipitating double diabetes, all being strong risk factors for the development of diabetic micro- and macrovascular disease [32]. The prevalence of comorbidities increased with duration of type 1 diabetes, and patients displayed diabetic nephropathy and neuropathy at a rate similar to that reported by previous studies [33].

This is to be regretted, as occurrence of diabetic complications can be minimized by aiming as closely as possible at benchmark values of diabetes care, as convincingly shown by the DCCT/EDIC study [34].

Significantly, in addition to its beneficial effects for the patients themselves, type 1 diabetes care at a DRC also comes with an economic advantage, as the costs per day amount to only 15 to 25% of that in standard hospitals (Table B in S1 File) frequently accepting the same patients with HbA_1c_ far above therapeutic target.

In addition, lowering HbA_1c_ somewhat below therapeutic target also provides some economic benefit per se, since patients sustaining HbA_1c_ levels <53 mmol/mol for >3 years require less financial support during this period (−$5,214.—) than those with HbA_1c_ ≥53 mmol/mol [35].

The limitations of this observational study, include its (i) single center observational nature, (ii) reliance on patient compliance with standardization at the DRC, and (iii) absence of control of patient adherence to treatment recommendations in their external environment after discharge, where they tend to relapse at a considerable rate [36]. Thus, the long-term benefit of intervention at a DRC needs to be answered in randomized multi-center clinical trials.

## Conclusion

We conclude that i) to meet goals of type 1 diabetes care insulin treatment, food intake and exercise have to be handled in concert and the respective recommendations implemented, ii) this need is helped by simple standardization of lifestyle, which easily also could be implemented at home, iii) compliance with rules of insulin treatment in T1D care cannot be replaced by arbitrary medication with add-on glucose-lowering drugs, and iv) costs of necessary patient education can be considerably reduced if offered at a DRC instead of acute hospitals.

## Supporting information

S1 File Table A of S1 File gives detailed results from general linear models assessing changes in vital and metabolic variables in response to 3 weeks at the DRC in patients with and without changes in insulin treatment modes. Table B summarizes costs of hospitalization depending on Austrian hospital types. Fig A depicts improvement of vital and metabolic variables in patients with and without changes in insulin treatment strategies.
(DOCX)
