# Supplementary material for: Type 1 diabetes care: Improvement by standardization in a diabetes rehabilitation clinic. An observational report
Source: PLoS One. 2018 Mar 12;13(3):e0194135. doi: 10.1371/journal.pone.0194135 (PMC5847233; doi:10.1371/journal.pone.0194135)
Supplement: S1 File — Table A of S1 File gives detailed results from general linear models assessing changes in vital and metabolic variables in response to 3 weeks at the DRC in patients with and without changes in insulin treatment modes. Table B summarizes costs of hospitalization depending on Austrian hospital types. Fig A depicts improvement of vital and metabolic variables in patients with and without changes in insulin treatment strategies. (DOCX) [file pone.0194135.s001.docx]

# Supplemental File

**Table A**

|  | | **Interaction**  **cohort x time** 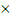 |
| --- | --- | --- |
| **BMI** | | F=0.810, p>0.05 |
| **Waist circumference** | | F=0.959, p>0.05 |
| **Blood pressure** | systolic | F=0.166, p>0.05 |
|  | diastolic | F=0.577, p>0.05 |
| **HbA_1c_** | | F=0.330, p>0.05 |
| **Fasting blood glucose** | | F=0.010, p>0.05 |
| **Total cholesterol** | | F=0.026, p>0.05 |
| **LDL cholesterol** | | F=0.068, p>0.05 |
| **Triglycerides** | | F=0.344, p>0.05 |
| **LDL/HDL ratio** | | F=0.104, p>0.05 |

Independence of changes in vital and metabolic variables in response to 3 weeks at the DRC from changes in insulin treatment mode in T1D-patients [N = 109] when analyzed by general linear models. Any significant interactions cohort x time would indicate a different HbA1c development in patients with strategy adaptations when compared to individuals without these changes.

**Table B**

|  | **Costs/day** [range] |
| --- | --- |
| **Hospitalization** |  |
| **- University hosp.** | € 1,145.-- |
| **- Acute** [non university] **hospital** | € 682.--  [594 – 816] |
| **- Acute hosp.,** ICU | € 1,813.--  [1,351 – 2,042] |
| **Rehabilitation Clinic** | € 131.-- |

Costs of hospitalization [€/d and patient; range] depending on hospital type in Austria. Data: Parlamentary records 891/AB XXV. GP, [www.parlament.gv.at](http://www.parlament.gv.at), and <https://www.akhwien.at/default.aspx?pid=789>

**Fig A**

**

**

Identical rate of improvement in vital and metabolic variables [Means ± SD] obtained in T1D patients with (●, N = 59) and without (○, N = 50) change in insulin treatment strategy during three weeks at the DRC [assessment by general linear models]
